# Incidence, predictors and severity of adverse events among whole blood donors

**DOI:** 10.1371/journal.pone.0179831

**Published:** 2017-07-21

**Authors:** Hamdan Almutairi, Mahmoud Salam, Abdulaziz Alajlan, Faisal Wani, Bushra Al-Shammari, Khaled Al-Surimi

**Affiliations:** 1 Department of Pathology and Laboratory Medicine, Ministry of National Guard Health Affairs, Riyadh, Saudi Arabia; 2 King Abdullah International Medical Research Center, King Saud bin Abdulaziz University for Health Sciences, Riyadh, Saudi Arabia; 3 College of Public Health and Health Informatics, King Saud bin Abdulaziz University for Health Sciences, Riyadh, Saudi Arabia; 4 Department of Primary Care and Public Health, School of Public Health, Imperial College London, United Kingdom; KSAU-HS, SAUDI ARABIA

## Abstract

**Background:**

Adverse events have been reported post blood donation. Donors might refrain from donating again due to such events which lowers the blood supply in collection centers.

**Aim:**

This study measured the incidence, predictors and severity of adverse events among donors of a single whole blood unit at one of the largest donation centers in Saudi Arabia.

**Methods:**

A retrospective cohort was conducted in 2015 to investigate the adverse events immediately post donation. Donor characteristics such as age, blood pressure, hemoglobin level, weight and history of donation were described and tested as potential risk predictors. Eligible blood donors were 18,936/24,634 (76.8%).

**Results:**

Incidence of adverse events found 1.1% (208 donors), of which 0.65% had mild symptoms (chills; nausea; pallor; dizziness; nervousness; headache), while 0.45% had severe symptoms (hypotension; convulsions; syncope; respiratory distress; emesis). Multiple logistic regression showed that, the incidence of adverse events was significantly higher among young age donors <30 years RR[95%CI] = 1.58[1.18–2.12], p < 0.002, higher hemoglobin levels RR[95%CI] = 1.30[1.15–1.46], lower weight donors <75kg RR[95%CI] = 1.71[1.29–2.27], p <0.001 and first time donors RR[95%CI] = 2.21[1.64–2.97], p < 0.001 compared to older age donors ≥30, lower hemoglobin levels, heavier weight donors ≥ 75, and previous donors, respectively. More severe adverse events were observed among older and heavier donors, previous donors, lower hemoglobin levels and hypertensive donors but with no statistical significance.

**Conclusion:**

Young blood donors, donors with lower weight and first time donors are at higher risk of contracting adverse events. Higher hemoglobin level is also a potential risk predictor of adverse events post whole blood donation.

## Introduction

Blood donation is considered to be one of the most valuable contributions of an individual to the community. Blood transfusion is vital and fundamental in medical practice since there is no efficient substitute to human blood when needed. The collection of whole blood is usually restricted to healthy donors, so ensuring the safety of blood donors is an essential factor to encourage them to donate and ensure their return in the near future [1]. Although, blood donation has relatively low risk rate and donors undergo meticulous screening for any contraindications prior donation, some adverse events arise occasionally during or after the process [2].

The incidence and severity of adverse events post blood donation has been reported in literature despite being relatively low. For instance, among 29,524 donations an overall 108 adverse events were reported (0.37%) in one study [2], while in another study it was (2.5%) [1]. Adverse events are generally reported as being vasovagal reactions (63.5%) or hematomas (35.0%) [1]. Predictors of adverse events among young blood donors were more prevalent in those with lower weight, first time donors and females [3]. A study conducted in Japan has reported that the risk of vasovagal reactions related to blood donation was associated with young age, low body mass index (BMI), high blood pressure, fast pulse rate, first-time donation, small circulating blood volume and short sleep time [4]. Other predictors were the feelings of distress and anxiety of blood withdrawal among first timers. In addition, adverse events were associated with hemoglobin levels as a potential risk predictor, but this has not been studied sufficiently.

An incidence of adverse event will definitely leave a negative impact on the retaining of blood donors. Donors will eventually refrain from coming back in the future to donate, which will in return lowers the blood supply in donation centers [2]. It has been noted that 9% of donors who experience an adverse event at their first donation did not return for the second one [5]. Despite being reported in such low rates, further investigations ought to be done to eliminate such unfortunate events as well as to promote donor safety and satisfaction. Although the screening criteria for donation are strict, adverse events do occur and vary in severity which raises a debate of whether such criteria need evidence based recommendations for adjusting its components. Last but not least, there was a need to shed light on such events as there is a scarcity of data about this issue in the Middle East and Gulf region.

The purpose of this study was to investigate the incidence, predictors and severity of adverse events among donors of a single whole blood unit at one of the largest blood donation center in Saudi Arabia. This was fulfilled by observing for any adverse event during or directly after donation, testing some donor characteristics to identify their risk predictors and evaluating the severity of these events.

## Materials and methods

### Study design

Retrospective cohort.

### Study setting

King Abdulaziz Medical City (KAMC) is a distinguished Joint Commission International (JCI) accredited tertiary health care facility established in 1983. KAMC is dedicated to provide health services to the military community of Saudi National Guards and their dependents. The blood donor center at KAMC was established in 1984 under the department of pathology and laboratory medicine and has been accredited by the College of American Pathologist (CAP) and American Association for Blood Banks (AABB), both since 1986. The center is operated by 24 technicians, 5 apheresis nurses and 1 physician, all directed by a laboratory director and a supervisor. Its current capacity has reached 16 blood donation chairs, for donating whole blood and apheresis. On annual basis, the blood donation center supplies around 26,000 blood units to the community of KAMC, the public and hospitals all over the kingdom upon request.

The measuring equipment in this setting were of the same brand and model throughout the study period, routinely tested and calibrated, and used as per the operational manual of the manufacturer. Quality control checkups are daily, monthly and annually performed for the machines to ensure their reliability. Proficiency testing, which is required by CAP, is performed biannually for the hemoglobin machine to assess its efficiency and accuracy. All donors are always advised to drink sufficient amount of fluids and eat snacks (readily available at the donation center) prior donation. The body position of donors is routinely set at semi-inclined degrees during blood withdrawal. After the donation is over and the needle is retracted, donors are instructed to press hard on the needle site and report any signs of blood leaking or hematomas. Post donation instructions and education are provided to donors.

### Study subjects

Between January and December 2015, all donors of whole blood units visiting the study setting were screened for eligibility to donate. These were healthy adults, with no active diseases or infections, had not recently undergone any surgery, tattoo, vaccination, or taken medications. Eligible donors were also those who haven't travelled to high risk countries or haven`t engaged in unprotected sexual relationships. In addition, the screening criteria required donors to have normal vital signs, that is oral temperature <37.5 degrees Celsius, heart rate 60–100 beats per minute, systolic blood pressure between 100–180 mmHg) and diastolic blood pressure between 60-100mmHg. High blood pressure doesn't contraindicate blood donation [6]. The donation center withdrew 10.5 ml of blood per kilogram of the donors’ body weight, and the minimum acceptable weight was 50kg. The acceptable range of hemoglobin (Hgb) level for donation was 12.5–18 g/dl. Each donation was accounted as a single study subject, with the presence of multiple donations from a single individual. Potential blood donors who didn't meet the previously mentioned donation eligibility criteria were deferred from donation. Eligible donors who consented for blood donation were accounted as study participants. In Saudi Arabia, blood donation is non-paid and voluntary practice favored by the community and health care services, cherished by a religious and tribally bound culture.

### Data collection

Screening donors and blood withdrawal was performed by a team of well-trained full-time employees (phlebotomists and apheresis nurses), all registered in the Saudi Commission for health specialties. This team reported to the supervisor of the donation center ensured that the donation was in compliance with the standard operational procedures and accrediting bodies. Annual verification of skills or competency assessment checklists are conducted to keep these employees updated as well as to refresh their knowledge and technical skills on using the measuring tools at the center. All blood center employees are certified in Basic Life Support issued by the American Heart Association. Accordingly, phlebotomists were competent in observing any adverse event during or post blood donation. The data were extracted from medical records and was comprised of:

1. Informed consent: name, national identification number, date/ time, contact information, and signature of by the donor.
2. Donors’ medical history and characteristics: gender, age (years) categorized as younger group (<30 years) and older group (≥30 years), gender, weight (kilograms) categorized as (<75 kg and ≥75kg) [7], temperature (degree Celsius), pulse rate (beats per minute), blood pressure (mmHg) categorized into two groups normal/ pre-hypertensive and hypertensive [8]. Hemoglobin level was categorized and presented in four groups (g/dl).
3. Adverse donor reaction: a list of observed and/or donor reported signs/symptoms that occurred during or immediately after donation at the donor center were recorded. Signs/symptoms were categorized as mild adverse events (chills, nausea, pallor, dizziness, nervousness or headache) and severe adverse events (hypotension, muscle contractions, convulsions, syncope, respiratory problems or emesis) as classified in literature [9–13]. In case the donor manifested both mild and severe signs/symptoms simultaneously, they were accounted as severe adverse events.

### Data management and analysis

SPSS statistical software (Version 23; SPSS Inc., USA) was used for data entry and analysis (S1 File). Sample characteristics and adverse events were presented in frequencies and percentages. Incidence was calculated by dividing the number of events within a group over the total number of donors in this group multiplied by 100. Bi-variate analysis using pearson’s chi-square test (χ2) was used for categorical data such as age groups, blood pressure category, Hgb levels, weight group and history of donation. A univariate analysis followed by multivariate analysis using a binary logistic regression was constructed to identify the risk predictors of adverse events and there severity. These risk predictors were selected accordingly as they have been commonly reported in literature to have various degrees of effect on the incidence and severity of adverse events post donation [3,4]. The adjusted relative risk (ARR) and its 95% confidence intervals (95%CI) were obtained. The P-value (P) was set significant < 0.05.

### Ethical consideration

The screening for donation eligibility criteria, vital signs, hemoglobin testing and phlebotomy is routinely performed process at the blood center. This was an observational study without altering the routine process. There was neither a usage of experimental items nor a testing of a new product. Data collectors were well-trained health care professionals. Patient privacy and confidentiality of data were secured. Donors were informed that their data will be used for multiple purposes such as generating quality indicators, improvement projects and research activities without revealing any donor identification. This study was approved by the institution review board (IRB) of the Ministry of National Guard, Riyadh, Saudi Arabia (SP 16/147).

## Results and discussion

### Blood donor characteristics

In 2015, a total of 24,634 potential blood donors visited the setting, yet 18,936 (76.8%) were eligible to donate one unit of whole blood (450±45 ml). Higher acceptance rates were significantly associated with males 18,676 (77.4%), older donors ≥30 years 10,315 (77.8%), heavier weight donors ≥75 kg 12,917 (96.6%), previous donors 9,926 (81.9%), normal/pre-hypertension category 14,860 (94.8%), compared to females 260 (49.9%), donor with age <30 years 8,621(75.6%), those with weight <75 kgs 6,019 (95.7%), first time donors 9,010 (71.9%), and hypertensive visitors 4,076 (94.3%), respectively.

The majority of donors who contracted adverse events were males (n = 205), while females were three. Their mean of age was 26.5±7.9 years. A total of 130 (70.7%) donors were <30 years of age and 78 (29.3%) were ≥30 years. Almost half 99 (47.6%) of the sample had weight <75 kgs and 109 (52.4%) had weight ≥75 kgs. The majority of sample were first time donors 144 (74.5%), while the rest 64 (25.5%) had previously donated whole blood. Normal and pre-hypertensive blood pressure was observed in 167 (80.3%) of the sample, while 41(19.7%) were classified as hypertensive.

### Incidence and risk predictors of adverse events

The incidence of adverse events among all donors was 208/18,936 (1.1%), of which the rate of mild events was 123/18,936 (0.65%) and severe event was 85/18,936 (0.45%). The incidence of adverse events was higher among donors <30 years 1.5% (p<0.001), donors with weight <75 kg 1.6% (p<0.001), first time donors 1.6% (p<0.001) compared to donors ≥30 years old (0.8%), weight ≥75 kg (0.8%), and previous donors (0.6%) respectively. Donors with higher hemoglobin levels >15 g/dl (1.3%) were significantly at higher risk to contract adverse events compared to lower hemoglobin levels Hgb 14.1–15 g/dl (0.7%), Hgb 13.1–14 g/dl (0.7%), and Hgb 12.5–13.0 g/dl, p = 0.005 (Table 1).

**Table 1:** Incidence of adverse events with donor characteristics.

	Sample	Adverse events	No adverse events
		n (%)	n (%)
	18,936	208 (1.1)	18,728(98.9)
**Gender**			
Male	18,676	205 (1.1)	18,471(98.9)
Female	260	3 (1.2)	257 (98.8)
		χ2 = 0.007, p = 0.931	
**Age (years)**			
<30	8,621	130 (1.5)	8491(98.5)
≥30	10,315	78 (0.8)	10,237(99.2)
		χ2 = 24.30, p<0.001*	
**Weight (Kg)**			
<75	6,019	99 (1.6)	5,920(98.4)
≥75	12,917	109 (0.8)	12,808(99.2)
		χ2 = 24.25, p<0.001*	
**Donation history**			
First time donor	9,010	144 (1.6)	8,866 (98.4)
Previous donor	9,926	64 (0.6)	9,862 (99.4)
		χ2 = 39.38, p<0.001*	
**Hemoglobin level (g/dl)**			
12.5–13.0	440	2 (0.5)	438 (99.5)
13.1–14.0	1,641	12 (0.7)	1,629 (99.3)
14.1–15.0	3,381	23 (0.7)	3,358 (99.3)
> 15	13,474	171 (1.3)	13,303 (98.7)
		χ2 = 12.77, p = 0.005*	
**Blood pressure**			
Normal/prehypertensive	14,860	167 (1.1)	14,693 (98.9)
Hypertension	4,076	41 (1.0)	4,035 (99.0)
		χ2 = 0.410, p = 0.522	

n: frequency,

%: percentage,

χ2: Chi-square,

p: p-value,

*: significance at <0.05, g/dl: gram per deciliter.

Univariate analysis followed by multiple logistic regression was constructed to adjust for all variables and control for any possible confounder to identify the significant risk predicator of adverse events. The incidence of adverse events was significantly higher among young age donors <30 years ARR = 1.58[1.18–2.12%], higher hemoglobin levels ARR = 1.30[1.15–1.46], lower weight donors <75kg ARR = 1.71[1.29–2.27%] and first time donors ARR = 2.21[1.64–2.97%] compared to older age donors ≥30, lower hemoglobin levels, heavier weight donors ≥ 75, and previous donors, p = 0.002, p<0.001, p<0.001 and p<0.001 respectively (Table 2).

**Table 2:** Significant predicators of adverse events.

	Univariate analysis		Multiple logistic analysis	
	RR [95%CI]	P-value	ARR [95%CI]	adj.P-value
**Age (years)**				
<30 *vs*. ≥30^0^	2.01 [1.52–2.66]	<0.001*	1.58 [1.18–2.12]	<0.002*
**Weight (Kg)**				
<75 *vs*. ≥75^0^	1.97 [1.49–2.58]	<0.001*	1.71 [1.29–2.27]	<0.001*
**Donation history**				
First time *vs*. Previous^0^	2.50 [1.86–3.36]	<0.001*	2.21 [1.64–2.97]	<0.001*
**Hemoglobin level**				
(g/dl)	1.28 [1.14–1.44]	<0.001*	1.30 [1.15–1.46]	<0.001*

RR: relative risk, CI: confidence interval, ARR: adjusted relative risk, adj: adjusted,

^0^: reference group, g/dl: gram per deciliter,

%: percentage,

*: significance at <0.05.

**Fig 1:**
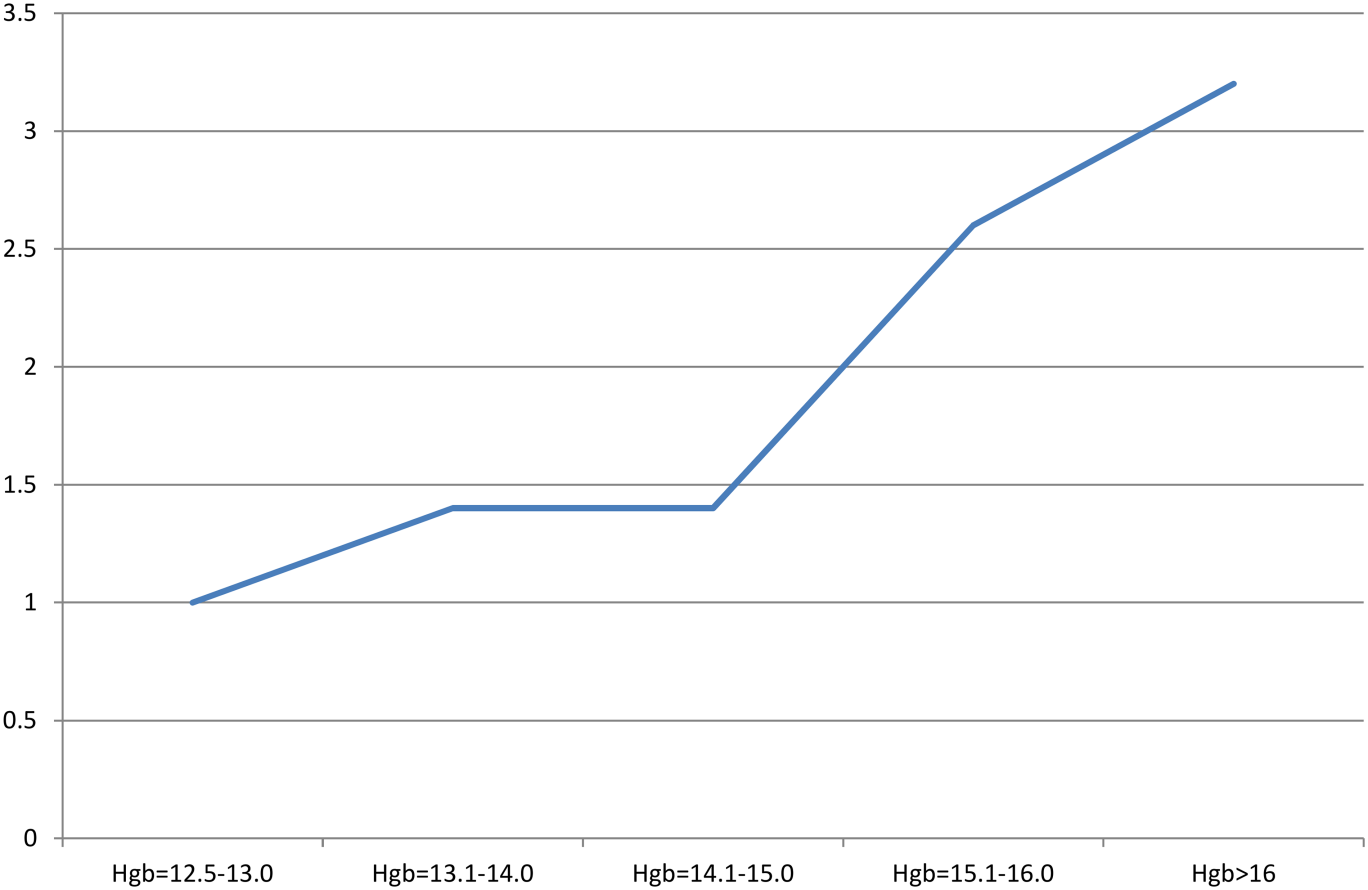
Relative risk curve of adverse events in relation with the hemoglobin levels.

As we can see also in the Fig 1 the incidence of adverse events was in a direct positive relationship with the level of hemoglobin among donors (Fig 1).

### Severity of adverse events

The leading mild adverse signs/symptoms among all donors were dizziness (84.6%) and pallor (75.5%), whereas the leading severe adverse events was hypotension (14.4%) and syncope (13.0%). One case of hematoma was observed, while no cases of allergic reactions were reported (Table 3).

**Table 3:** Frequency of mild and severe adverse events post blood donation.

Adverse events	n(%)*
**Mild**	
Chills	4 (1.9)
Nausea	20(9.6)
Pallor	157 (75.5)
Dizziness	176(84.6)
Nervousness	7(3.4)
Headache	2(1.0)
**Severe**	
Hypotension	30 (14.4)
Muscle contractions and convulsions	12(5.8)
Syncope	27(13.0)
Respiratory problems	5(2.4)
Emesis	14(6.7)
Diaphoresis	19(9.1)

n: frequency,

%: percentage,

*: non-mutually exclusive

Mild adverse events were observed in 123 (59.1%) of the subset sample with adverse events (208), while severe adverse events were observed in 85 (40.9%). The occurrence of severe adverse events was observed in 40% of donors < 30 years old, while in 42.3% of donors ≥30 years old. Half of the donors with hemoglobin level 12.5–13.0 g/dl endured severe adverse events (50%), while the least rate of severe events (40.9)% was observed in donors with Hgb level >15 g/dl. Normal and pre-hypertensive donors were at lower risk to endure severe adverse events (38.9%). Donors with weight <75 kgs had 40.4% chance of enduring severe adverse outcomes. Those with previous history of blood donation had higher chance 48.4% of enduring severe events. Bivariate analyses revealed no statistical significance between these various exposures (age category, hemoglobin levels, blood pressure, weight, donation history), Table 4.

**Table 4:** Severity of adverse events post blood donation with its significant risk predictors.

	Mild Side effects	Severe Side effects
	n (%)	n (%)
	123 (59.1)	85(40.9)
**Incidence**	**0.65%**	**0.45%**
**Age (years)**		
<30	78(60.0)	52(40.0)
≥30	45(57.7)	33(42.3)
	χ2 = 0.107, p = 0.743	
**Weight (Kilogram)**		
<75	59(59.6)	40(40.4)
≥75	64(58.7)	45(41.3)
	χ2 = 0.017, p = 0.897	
**Donation history**		
First time donor	90(62.5)	54(37.5)
Previous donor	33(51.6)	31(48.4)
	χ2 = 2.193, p = 0.139	
**Hemoglobin level (g/dl)**		
12.5–13.0	1 (50)	1 (50)
13.1–14.0	8 (66.7)	4 (33.3)
14.1–15.0	13 (56.5)	10 (43.5)
Above 15	101 (59.1)	70 (40.9)
	χ2 = 0.416, df = 3, p = 0.937	
**Blood pressure**		
Normal/prehypertension	102 (61.1)	65 (38.9)
Hypertension	21(51.2)	20 (48.8)
	χ2 = 1.324, p = 0.250	

n: frequency,

χ2: Chi-square,

p: p-value, x: mean, df: degree of freedom, SD: standard deviation,

*: significance at <0.05.

### Discussion

Adverse events post the donation of whole blood seems inevitable. The incidence in this study was 1.1% in a one year study period, which was less than studies conducted in three large blood centers that reported a total incidence of 16,129/724,861(2.3%) [14]. The American Red Cross has reported an incidence of vasovagal reactions among 435 cases per 10,000 blood donations [15]. However, two studies noted a lower incidence of vasovagal reactions in 0.2% [9] and 0.59% [16]. Another study conducted in the region found that higher incidence of adverse events 342 (2.8%) were observed in a total of 11,941 donations (whole blood and apheresis), of which 2.5% were associated with whole blood donation [17]. The methodological difference existing between these studies could be one of the possible explanations for reporting different incidence of such adverse events; however the incidence of adverse events post blood donation remains an important quality indicator required by accrediting bodies for any donor center that should be reported annually and benchmarked locally or internationally.

The etiology of such rare adverse events (1.1%) within a study population of 18,936 who all passed the preset donation criteria remains unclear. Blood donors passed through an assessment of vital signs and testing of hemoglobin levels using standardized measuring equipment. All study participants were previously healthy and had no contradictory criteria for donation. All laboratory tests conducted later on a small blood sample retrieved from blood units cleared any infectious diseases or anemia. Studies have observed a significant effect of offering fluids and snacks before starting the donation on the development of adverse events [18–19]. Despite the fact that the donation center readily provided snacks prior and after donation, authors suspected a lack of compliance from the donors’ side. Therefore, the approach of this study was to test some donor characteristics with the severity of such incidents, rather than merely their incidence.

Although higher incidence of adverse events was observed in donors with higher hemoglobin levels, the severity of adverse events was in an inconsistent relationship with the hemoglobin levels. One Japanese study recently noted that elevated hemoglobin and serum protein levels were indicators of higher rates of vasovagal reactions, which was similar to the findings in this study [20]. Although all study participants enrolled in this study had Hgb levels above the minimum eligibility criteria for donation (12.5 g/dl), surprisingly donors at the upper end of Hgb levels (>15 g/dl) were at the highest risk of contracting adverse events which raises a point of whether to set both a low and a high marginal limit of donation criteria to ensure donor safety. It is worth mentioning that a study noted that increased hematocrit levels were closely associated with higher rates of vasovagal reactions [14]. An unpublished study in a regional university hospital reported an incidence of adverse events (2.8%), yet no testing was done for the significant risk predicators of these reactions [17].

The weight of participants which is a less reflective indicator than the body mass index (BMI) was also a significant contributing factor of severe adverse events. One study noted that as the weight of a donor increased from 45 kg to more than 80 kg, the risk of developing of adverse reactions decreased (p<0.001) [21]. The volume of blood collected in this setting was 450±45 ml and the minimum volume to donor weight adopted was 10.5ml/kg. The cut off weight in literature was investigated at lower weights (60kg) [21] which again proved that lower weight is a significant predictor of adverse events. Other studies reported that the risk of adverse events were twice expected in donors weighing <54kg [22]. In addition, the risk predictors of adverse events in this study (younger age groups, lower weight donors, those who were first time donors) were comparable to findings published in another study [3]. A survey found that a total of 32% of first-time and 14% of previous donors complained of severe adverse events [23]. However, this study didn’t reveal a significant relationship between being a single versus multiple donations on the severity of adverse events.

### Limitations

This study had some limitations. Delayed adverse events associated with blood donation such as hematomas that usually occur after leaving the blood donation center were not investigated. Investigators also suspected an under reporting of some adverse events, which might be perceived by phlebotomists and nurses as an indicator of their malpractice or incompetence. In addition, although the blood donor center has policies and procedures regarding the recognition, handling and managing of such adverse events, a clear distinction between the severities of adverse events was not established. In other words, categorizing these events is subjective to the assessment of medical technicians and nurses. Other risk predicators might be present, such as the lack of sleep, insufficient intake of meals/fluids prior donation, needle phobia or hemophobia especially among first time donors, yet this study didn’t include and test them in this study setting. Authors suspected that the self-reporting of these potential confounders might have been subject to some inaccuracies or recall biases from the donors’ side.

### Recommendations

All nurses and phlebotomists working in donation centers should be aware of the risk predicators highlighted in this study, so that adverse events are anticipated prior donation. Visual illustration of the relative risk curve can be posted in donation centers to clarify the relationship between hemoglobin levels and the incidence of adverse events. Revised policies and guidelines should be established at blood donation centers to ensure the staff is compliant. Other strategies to reduce the incidences of adverse events among donors with risk predictors (young age, low weight) should be generated and implemented. Adverse events should be recorded and monitored monthly so that this quality indicator is benchmarked with local and international standards. Further interventional approaches might be taken to lower or eliminate such unfortunate incidences.

## Conclusion

The incidence of adverse events associated with whole blood donation in this study is slightly lower than international figures. These adverse events are higher among young age donors, with lower weights, with higher hemoglobin levels, and first time donors. The severity of the adverse event ranges between mild and severe with no statistically significant difference documented.

**Key points**

- Adverse events, such as vasovagal symptoms, have been observed post blood donation.
- Donors might refrain from donating again due to such events which lowers the blood supplies in collection centers.
- Incidence of adverse events is higher among young age donors, those with lower weight, those with higher hemoglobin levels, and first time donors.
- Severity of adverse events increases with lower hemoglobin levels.
- Donation centers should be aware of the risk predicators highlighted in this study, so that adverse events are anticipated prior donation.

## Supporting information

S1 File SPSS file. (SAV)
